# Proximal Post-Curing Improves Microhardness of Class-II Resin Composite Restorations

**DOI:** 10.3390/jfb17080383

**Published:** 2026-08-04

**Authors:** Sittah Reinhard, Sebastian Soliman, Britta Hahn, Roland Frankenberger

**Affiliations:** 1 Department of Operative Dentistry, Endodontics, and Pediatric Dentistry, University of Marburg and University Medical Center Giessen and Marburg, Campus Marburg, Georg Voigt Str. 3, D-35039 Marburg, Germany; 2 Department of Conservative Dentistry and Periodontology, Center of Dental Traumatology, University Hospital of Würzburg, Pleicherwall 2, D-97070 Würzburg, Germany; soliman_s@ukw.de (S.S.); hahn_b1@ukw.de (B.H.)

**Keywords:** resin-based composites, light-curing, proximal post-curing, longevity, microhardness, photopolymerization

## Abstract

**Aim**: This in vitro study investigated the effect of additional proximal post-curing on the microhardness of Class-II resin composite restorations. **Methods**: Thirty-two extracted human third molars received narrow (N) or wide (W) MO Class-II cavities with cervical margins extending below the cemento-enamel junction. Cavities were restored with Venus Diamond using an adhesive protocol and incremental layering. Specimens were assigned to four groups: N.O, N.OT, W.O, W.OT, representing cavity width (N/W) and curing protocol (O: occlusal light curing only, OT: occlusal plus proximal post-curing after matrix band removal). After 7 days of storage in artificial saliva, restorations were sectioned into three vertical planes, and Vickers microhardness (HV2/30 s) was measured. **Results**: The highest overall microhardness was observed in Group W.OT (81.35 HV2), whereas Group N.O showed the lowest values (73.14 HV2) (*p* < 0.05). Proximal post-curing significantly increased microhardness, with Group N.OT (78.34 HV2) exceeding both occlusal-only groups (W.O: 74.68 HV2; *p* < 0.05). In all groups, microhardness decreased with increasing restoration depth (*p* < 0.05), although this reduction was less pronounced after post-curing. **Conclusions**: Within the limitations of this in vitro study, proximal post-curing significantly increased polymerization depth and microhardness of Class-II resin composite restorations.

## 1. Introduction

Despite effective preventive measures, dental caries remains the most common noncommunicable oral disease worldwide [1]. Its prevalence is generally above average among socioeconomically disadvantaged population groups [2,3,4]. This underscores the need for effective, early, and affordable restorative treatment options for patients.

Direct resin-based composite restorations (RBC) have become more popular than amalgam and glass ionomer cement because of esthetic requirements and minimally invasive concepts. Furthermore, they also have technical advantages over ceramic restorations [5,6,7,8,9,10,11]. They allow for tooth-preserving preparation and single-visit treatment, which is more cost-effective than ceramic restorations. Nevertheless, both secondary caries and filling fractures have been reported to be the most frequent complications [12,13,14]. RBCs are technique-sensitive, and their success largely depends on the user [13]. While research into improving composite restorations has mainly focused on improving adhesive systems and chemical compositions of resin composites, research into light-curing techniques has only been given greater consideration in recent years, even though they are increasingly considered a critical determinant of the long-term success of light-cured restorations [14]. Resin composites are placed in the cavity in a plastic state and cure through light exposure via radical photopolymerization. Photoinitiators, mostly camphorquinone, are split into radicals upon irradiation with visible light. The radicals can attack monomers, triggering a chain reaction that leads to the formation of a polymer chain. The degree of conversion (DoC) that can be achieved and the cross-linking structure significantly determine the mechanical properties and biocompatibility of the restoration [15,16]. The higher the DoC, the higher the resulting hardness of the composite. Hardness is therefore considered an indirect indicator of the DoC [17]. If resin composites do not cure completely, areas of lower hardness are formed [16,18]. These can be washed out, form vulnerable sites for secondary caries, and increase fracture risk [16,19,20]. Furthermore, insufficiently polymerized composites contain higher amounts of residual monomers. These can detach from the composite and cause gingival and pulp irritation, or even irreversible pulpal damage [18,21,22,23]. Critical areas are regions with limited light accessibility, especially deep or undercut areas in Class-II cavities [20,24,25,26,27]. At the cervical proximal margin, increased distance, absorption and scattering in enamel and dentin, as well as temporary shielding by metal matrices, can lead to reduced polymerization of the material and thus to reduced conversion rates [24,28]. As a clinical countermeasure, transdental post-curing performed proximally after removal of the metal matrix is recommended in order to increase irradiation of these critical areas [29]. This contrasts with the manufacturer’s specifications for modern high-performance polymerization lamps, which promise sufficient curing even at greater distances. Equally contradictory are studies showing a general reduction in light transmission through dental hard tissues, such as dentin and enamel. This reduction is directly associated with a lower polymerization rate [30,31,32,33]. Although proximal transdental post-curing after matrix removal is frequently recommended in clinical teaching and restorative practice to compensate for reduced light exposure at the cervical proximal margin, this recommendation appears to be based primarily on theoretical considerations rather than direct experimental evidence. To the best of our knowledge, no study has specifically investigated whether this clinically recommended procedure improves polymerization in the cervical proximal region and deeper areas of Class-II RBCs compared to conventional occlusal curing alone. Consequently, it remains unclear whether proximal transdental post-curing provides a significant improvement in polymerization in these anatomically critical regions. Addressing this question is clinically relevant because a higher degree of conversion in these areas may enhance the mechanical properties and integrity of the restoration while reducing residual monomer elution. These effects may, in turn, increase fracture resistance and marginal degradation, decrease the risk of secondary caries, and ultimately enhance the longevity of Class-II RBCs. If effective, proximal transdental post-curing could therefore represent a simple modification of the clinical workflow without increasing treatment complexity or requiring changes in restorative materials. Therefore, this study aims to determine the influence of corresponding post-curing procedures after removal of the metal matrix on Vickers microhardness (HV) in critical areas of Class-II composite restorations.

## 2. Materials and Methods

### 2.1. Specimen Preparation

**Figure 1:**
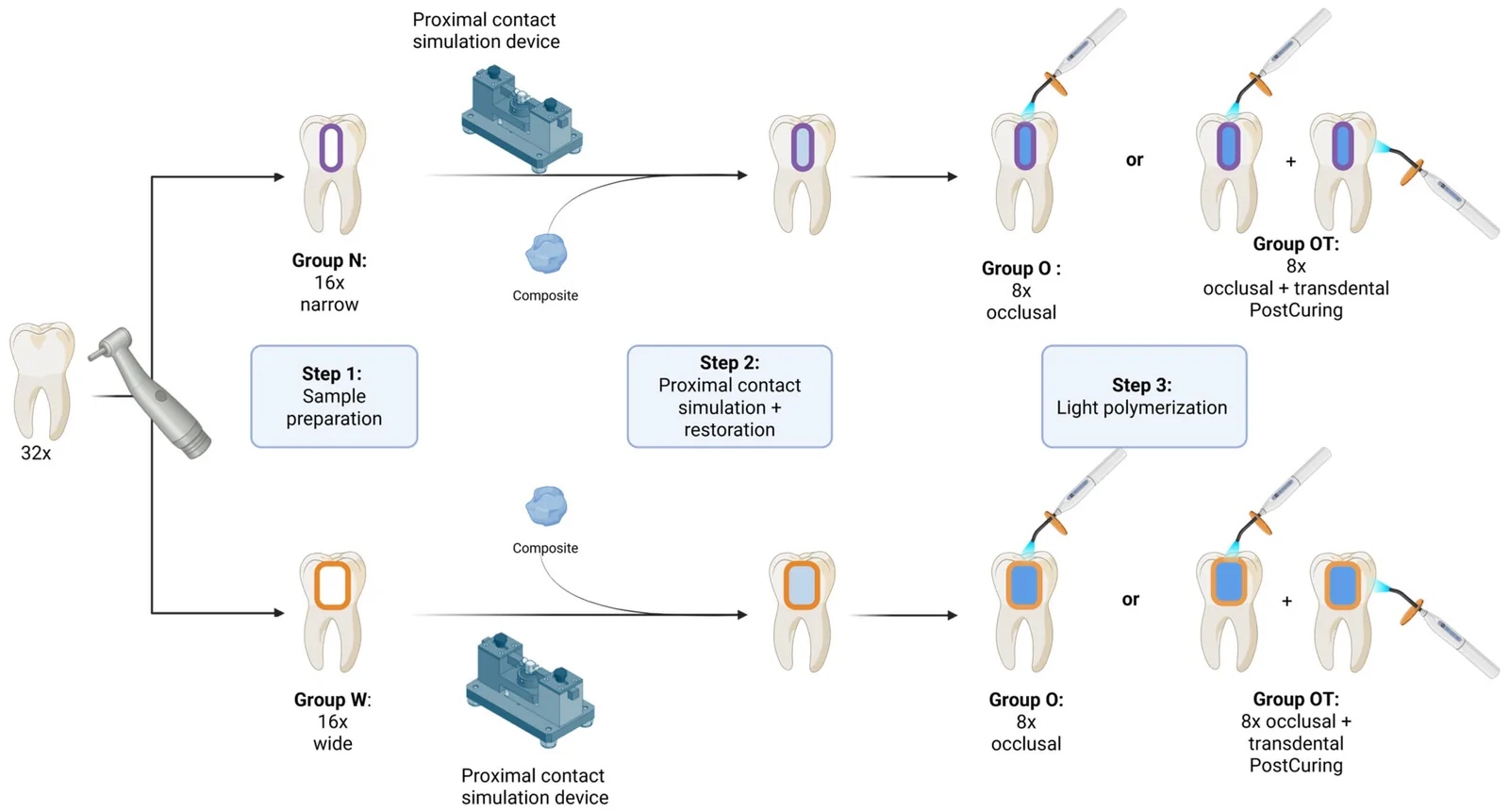
Sample preparation workflow. Step 1: Preparation of 32 teeth, 16 in Group N (cavity dimensions: 2.5 × 7 × 5 mm) and 16 in Group W (cavity dimensions: 4.5 × 7 × 5 mm). Step 2: Clamping the sample teeth in a proximal contact simulation device and performing restorative treatment using composite material. Step 3: Division of each group into two subgroups, followed by light curing of the restorations according to subgroup assignment: Subgroup O: occlusal light curing only; subgroup OT: occlusal light curing with additional transdental light curing (post-curing). Created in BioRender. Reinhard, S. (2026) https://BioRender.com/fqjxl2y.

Extracted human teeth were obtained from an IRB-certified biobank. The study was additionally approved by the local ethics committee (approval number: 25-342 BO). Thirty-two caries-free extracted human third molars were used. Based on our calculation for pairwise comparison (α = 0.05, power = 0.8, SD 10–15%, effect size 20–25 percentage points) PASS Software 26.0.4 (NCSS, LLC, Kaysville, UT, USA) computed a required sample size of *n* = 8 samples per group. Immediately after extraction, residual tissue was removed, and the teeth were stored for up to two months in a 0.9% isotonic NaCl solution containing 0.001% sodium azide for disinfection and preservation. Natural human teeth were used to closely resemble the clinical conditions, as enamel and dentin have unique optical properties, which cannot be adequately reproduced by artificial materials. Specimens were selected to have comparable overall morphology. Prior to specimen preparation, teeth meeting the inclusion criteria were evenly and randomly allocated into two experimental groups. A mesial Class-II cavity was prepared in all teeth, the width of which differed according to group assignment (Group N: narrow cavity, Group W: wide cavity) (Figure 1: Step 1). Cavities were prepared under continuous water cooling (50 mL/min) using cylindrical diamond burs with a parallel step (No. 6837, No. 8837, Komet Dental, Lemgo, Germany) at the maximum speed of a high-speed contra-angle handpiece (200,000 rpm) (EXPERTmatic LUX E25 L, Kavo Dental, Biberach, Germany). Group N received cavities with dimensions of 2.5 mm × 7 mm × 5 mm, while Group W received cavities with dimensions of 4.5 mm × 7 mm × 5 mm to simulate the impact of proximal post-curing on different cavity sizes. In both groups, proximal cavity margins were deliberately cut just below the cemento-enamel junction (CEJ) to simulate subgingival restoration margins. After preparation, the cavity dimensions were checked using a digital precision caliper (M240.016.1) from METAV Werkzeuge (Emmerich, Germany). Proximal wall thickness was not individually standardized across specimens, reflecting the natural anatomical variability encountered in clinical practice. Until the restorative treatment was performed, all tooth specimens were stored in artificial saliva according to Fusayama–Meyer (0.4 g/L NaCl, 0.69 g/L NaH_2_PO_4_, 0.4 g/L KCl, 0.906 g/L CaCl_2_, 1 g/L urea, 0.005 g/L Na_2_S, pH = 4.9).

### 2.2. Proximal Contact Simulator

**Figure 2:**
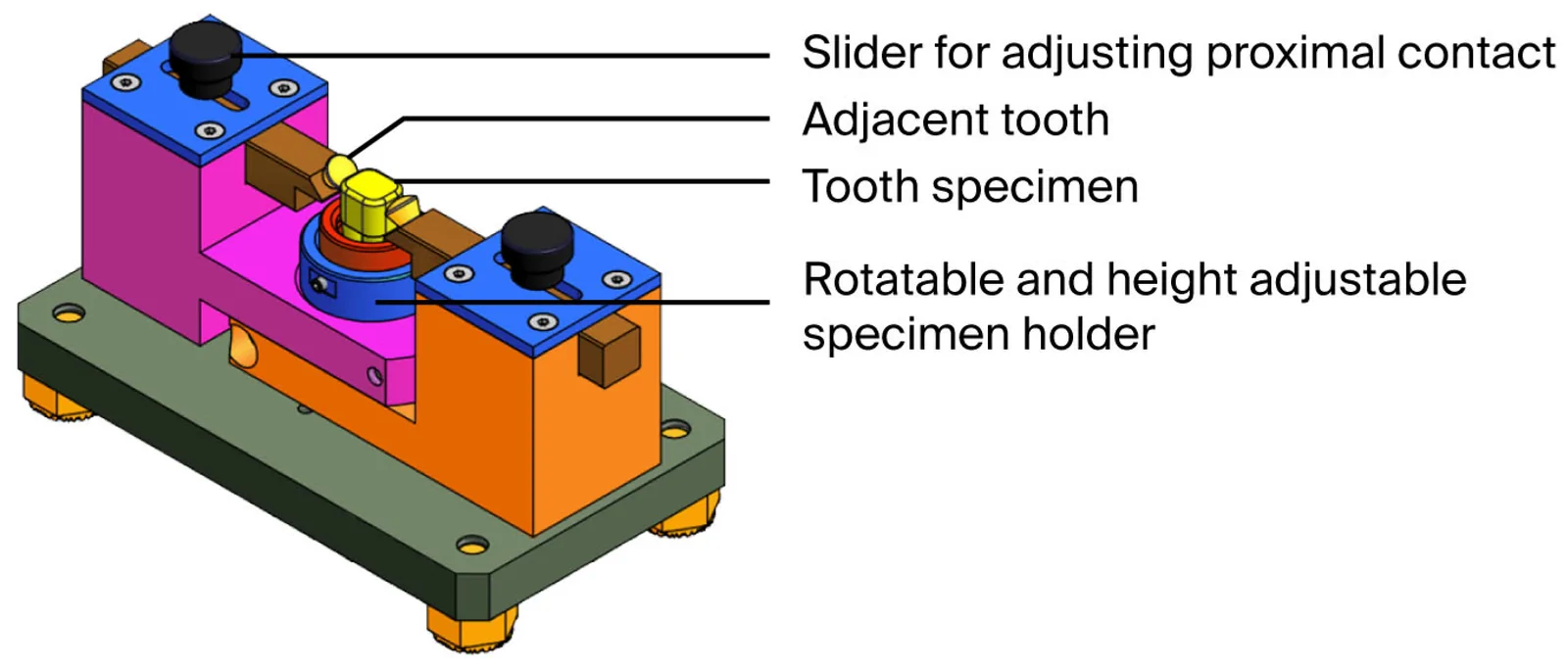
Proximal contact simulation device. A sample tooth (yellow) is embedded in a cylindrical holder (red). The carrier can be clamped in a matrix (blue) with adjustable height and 360° alignment. Sliders (brown) clamped to the side blocks (pink and orange) can be used to attach teeth (yellow) that simulate the proximal contact to the sample tooth as adjacent teeth.

To simulate clinically realistic proximal conditions, the samples were clamped into a proximal contact simulation device (Figure 1, Step 2), developed in collaboration with Schneider Metallbau GmbH (Kastellaun, Germany) and based on Soliman et al. [34] (Figure 2).

Each tooth was embedded in a central cylindrical sample holder using Impression Compound Green (KerrHawe SA, Bioggio, Switzerland), with the cervical cavity margin positioned 2 mm above the wax margin and the sample holder. Subsequently, the sample holder was clamped into the proximal contact device at an adjustable height and aligned with the neighboring tooth by rotation. An uncavitated molar was fixed on a movable slide to serve as the proximal adjacent tooth. The same natural tooth was used as the adjacent tooth for all specimens and was positioned at a defined distance and orientation relative to the sample tooth. The cusp tips of both teeth were aligned at the same height, with the vestibular and lingual surfaces parallel to each other. The interproximal distance was chosen so that there would have been firm proximal contact in an uncavitated state. In Group N, the proximal contact between the sample tooth and the adjacent tooth was only partially eliminated, whereas in Group W, the wide cavity geometry resulted in complete loss of proximal contact. Restoration of proximal contour and contact point was achieved using a Palodent V3 partial matrix (Dentsply Sirona Germany, Bensheim, Germany) in accordance with standard clinical practice.

### 2.3. Restoration and Light Curing

For adhesive pretreatment, all sample teeth were conditioned with phosphoric acid using the etch-and-rinse technique and then prepared with an adhesive system. For etching, phosphoric acid (DeTrey Conditioner 36, Dentsply Sirona Germany, Bensheim, Germany) was first applied to the enamel for 20 s and subsequently to the entire cavity for an additional 10 s. This resulted in a total etching time of 30 s for enamel and 10 s for dentin before thorough rinsing. After careful drying, a light curing universal adhesive system (Prime&Bond active, Dentsply Sirona, Konstanz, Germany) was applied. The adhesive was actively applied to both dentin and enamel for 20 s using a microbrush with light application pressure according to the manufacturer’s instructions, followed by air drying and light curing. Light curing was performed for 10 s using a light-emitting diode (LED) polymerization unit with an output of 1250 mW/cm^2^ (SmartLite Pro, Dentsply Sirona, Bensheim, Germany) with the output having been continuously monitored.

Restorative treatment was performed using the light-curing nanohybrid composite Venus Diamond shade A3 (Kulzer, Hanau, Germany) (Figure 1, Step 2). A 1 mm thin layer of flowable composite (Venus Diamond Flow, Shade A3, Kulzer, Hanau, Germany) was applied as a lining to initially seal the proximal cervical area and subsequently light cured for 20 s using the SmartLite Pro polymerization lamp (Dentsply Sirona, Bensheim, Germany). The packable composite was then applied in horizontal increments with a maximum layer thickness of 2 mm. This standardized horizontal layering technique was deliberately chosen to minimize procedural variability across specimens and to isolate the effect of the curing protocol on microhardness. It is consistent with protocols used in previous in vitro studies of Class-II composite restorations [35]. Each increment was evenly applied to the cavity and adapted using a ball and flat packer (Carl Martin, Solingen, Germany). Increment thickness was verified with a millimeter probe (HuFriedy, Frankfurt am Main, Germany) prior to light curing. Each increment was light cured individually (Figure 1, Step 3). A total of three incremental layers were placed, with the final layer finished flush with the height of the marginal ridge. The proximal contact was shaped according to the individual anatomy of each sample tooth. To achieve the most physiological contact point possible, the matrix was specifically adapted to the adjacent tooth during placement of the second or third increment.

All composite layers were polymerized using the high-performance SmartLite Pro LED lamp (Dentsply Sirona, Bensheim, Germany), which emits light primarily in the 450–480 nm range, corresponding to the absorption spectrum of camphorquinone [36]. Polymerization was performed incrementally for 20 s with the light guide positioned perpendicular to the cavity surface. The top increment was not touched by the light guide during polymerization.

Within each main group (W or N), half of the 16 sample teeth were allocated to subgroup O (purely occlusal polymerization) or subgroup OT (occlusal and additional transdental polymerization). In subgroup OT, all samples underwent additional proximal transdental post-polymerization following occlusal polymerization and removal of the metal matrix (Figure 1, Step 3). The polymerization lamp was positioned with its 10 mm light exit window aligned horizontally and directed towards the proximal space between the sample tooth and the adjacent tooth. In Group N, this alignment enabled indirect re-irradiation of the restoration through the dental hard tissue. In Group W, the more open proximal cavity geometry additionally allowed direct exposure of the restoration surface. Post-polymerization was performed both buccally and lingually. The lamp was held in direct contact with the tooth surface and post-polymerization was performed for 20 s on each side.

### 2.4. Cutting of Specimens

After polymerization, the sample teeth were stored for 7 days in artificial saliva according to Fusayama–Meyer at 37 °C and protected from light. All further steps were performed under a light protection box to strictly exclude light with wavelengths of more than 400 nm. The sample teeth were initially embedded in cylindrical molds using Technovit (Kulzer, Hanau, Germany). Subsequently, the embedded specimens were cut into thin slices using a precision cutting saw (IsoMet 1000 Precision Saw, Buehler, Leinfelden-Echterdingen, Germany) at 300 rpm under continuous water cooling.

**Figure 3:**
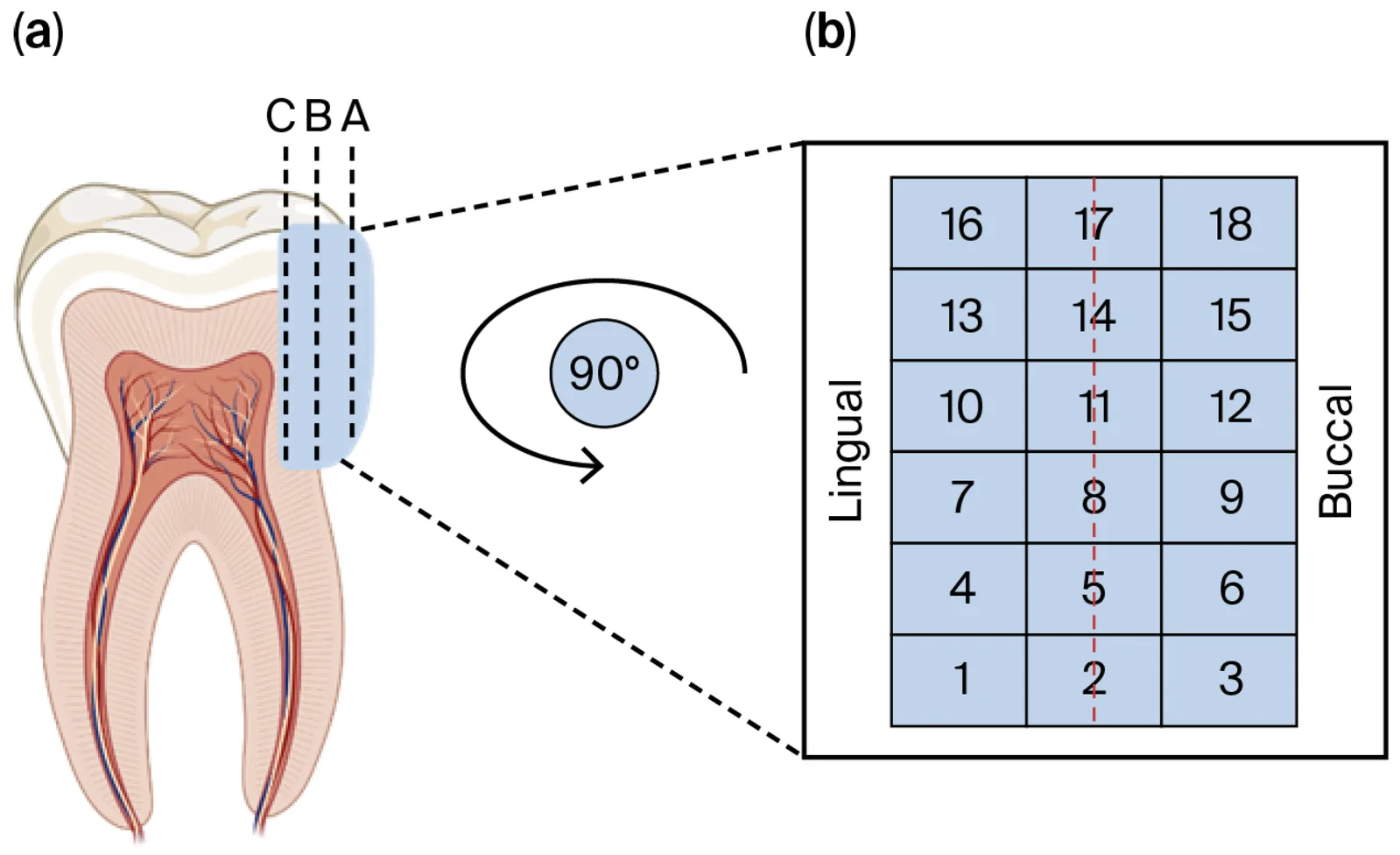
Section planes and measurement grid. (**a**) shows the section planes through the filling in mesiodistal alignment. Plane A: outermost plane close to the proximal contact; Plane B: central plane running through the center of the filling; Plane C: innermost plane running close to the pulp. (**b**) Planes rotated by 90°, frontal view of one plane, representation of the measurement grid with 18 measurement points on each plane in a lingual-buccal orientation. Central perpendicular line through the measurement grid shown in red. Created in BioRender. Reinhard, S. (2026) https://BioRender.com/lhyd841.

Each sample was sectioned by three parallel cuts at 2 mm intervals using a 0.4 mm-thick saw blade, resulting in disks with a uniform thickness of 1.6 mm. The cutting planes were oriented vertically in a bucco-lingual direction through the entire longitudinal axis of the sample tooth and the composite filling, resulting in completely proximal-oriented cross-sections. Three distinct planes were defined (Figure 3a): (A) the outermost plane adjacent to the proximal contact area; (B) the central plane passing through the center of the restoration; and (C) the innermost plane located closest to the pulp. The resulting saw-cut disks were subsequently stored in artificial saliva according to Fusayama–Meyer at 37 °C, protected from light until hardness measurements were performed.

### 2.5. Vickers Hardness Measurement

Microhardness was used as an indirect measure of the point-specific DoC of the composite specimens. To enable standardized assessment of the hardness distribution, each of the three defined filling levels (Plane A, B and C) was overlaid with a grid consisting of 3 columns and 6 rows (3 × 6 fields) (Figure 3b). Within this grid, measurements were taken horizontally from the lingual to the buccal aspect. Measurement point 1 marked the most crestal lingual area, whereas measurement point 18 marked the most coronal buccal area of the respective level. The intermediate measurement points were positioned on a marked perpendicular line passing through the center of the restoration.

To minimize measurement artifacts caused by the edge effects, a distance of 0.5 mm was maintained between the outer margin of the filling and the outermost measurement points. To prevent measurement distortion due to material compaction, a minimum distance of 2.5 times the indentation diagonal was maintained between the individual measuring points. Vickers hardness was determined using a microhardness tester (type 3212.000, ZwickRoell, Ulm, Germany) under a constant test force of 2 kg applied for a dwell time of 30 s. The resulting Vickers indentations were measured under 20× magnification. All Vickers hardness measurements were performed by a single, calibrated examiner. Prior to the study, the examiner underwent training measurements on pilot specimens and was calibrated against a reference value established by a second, experienced examiner. The intraclass correlation coefficient (ICC) was computed and demonstrated good reliability for Vickers hardness (ICC = 0.93, 95% CI: 0.82–0.98). Measurements were additionally performed in accordance with the manufacturer’s standardized protocol for the microhardness tester (type 3212.000, Zwick Roell, Ulm, Germany). All measurement data were recorded using the testXpert V12.5 evaluation software and documented in a digital test report. To visualize the hardness distribution within each filling level, the mean values of the 18 measurement points on each level were presented as heat maps, with a color scale ranging from red (low hardness values) to green (high hardness values).

### 2.6. Statistical Appraisal

Statistical analysis was performed using SPSS 17 (SPSS Inc., Chicago, IL, USA). After verifying normal distribution using the Kolmogorov–Smirnov test, significant changes between groups were calculated using two-way ANOVA and Tukey–Kramer tests at a significance level of 5%.

## 3. Results

### 3.1. Plane A

**Figure 4:**
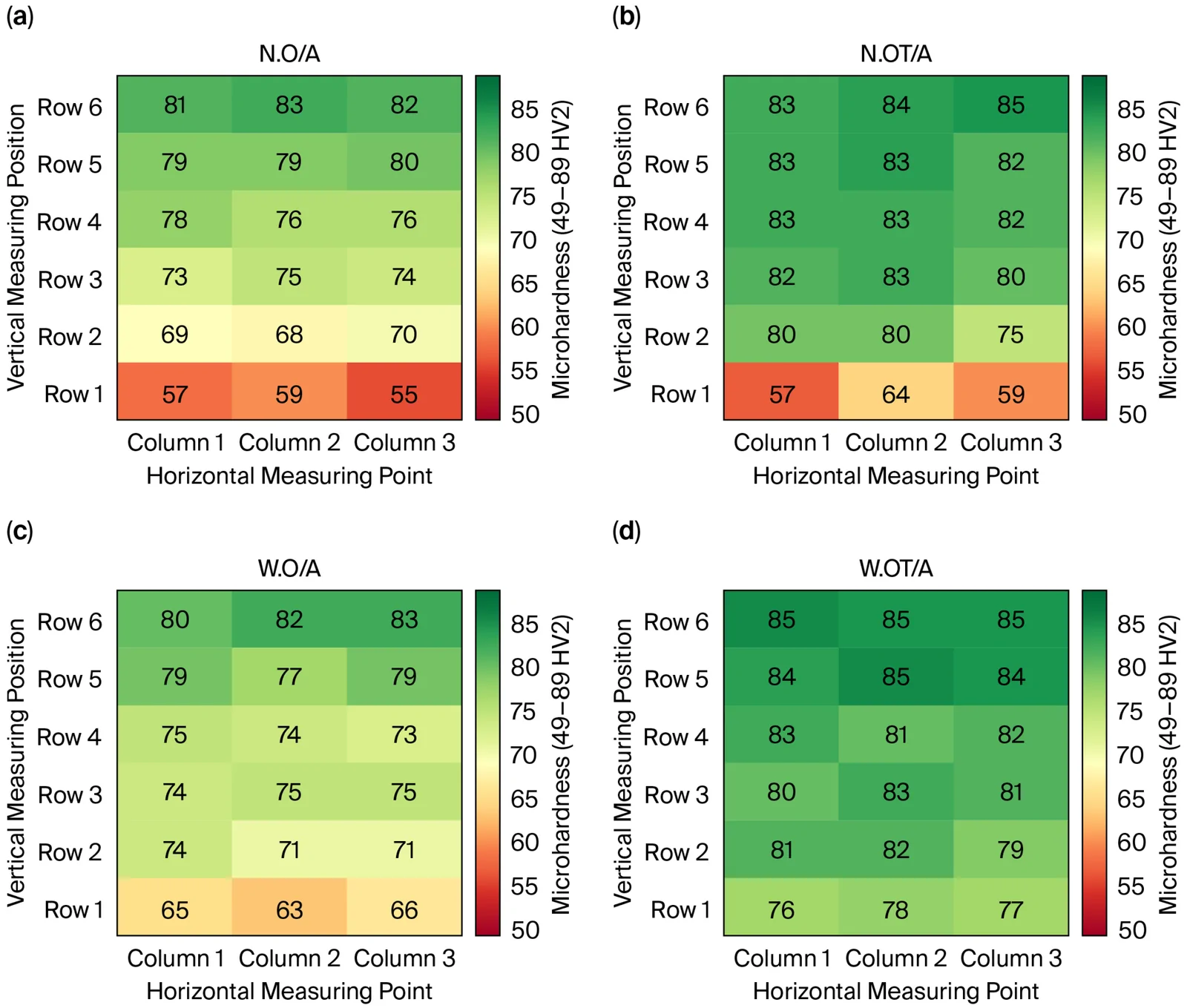
Heatmaps of mean Vickers microhardness (HV2) in the outermost Plane A for the four experimental groups ((**a**): N.O, (**b**): N.OT, (**c**): W.O, (**d**): W.OT). Rows represent the vertical measuring positions from the occlusal region (Row 1) to the crestal region (Row 6). Columns represent the horizontal measuring positions within the restoration. Values displayed within each cell correspond to the mean microhardness (HV2) measured at this location. Color reflects microhardness, with red color on the lower spectrum and green colors on the higher spectrum.

In the outermost Plane A, higher microhardness values were observed in the transdentally cured groups (OT) compared with the occlusally cured groups (O) (Figure 4).

All groups showed decreasing microhardness values from the occlusal (Row 1) to the crestal region (Row 6). The difference ΔHV(o,c) between the occlusal and crestal rows was 24,7 HV2 in N.O (82.0 vs. 57.3 HV2) and 17.3 HV2 in W.O (81.8 vs. 64.5 HV2). In the OT groups, the gradient was reduced, particularly in W.OT, where ΔHV(o,c) was 8.0 HV2 (85.0 vs. 77.0 HV2) and was significantly lower than in the respective W.O group (*p* < 0.05). N.OT showed an intermediate decrease in hardness of 22.5 HV2 (82.3 vs. 59.8 HV2).

In the central areas of the restorations (row 3 and 4), the OT groups consistently showed higher microhardness values than the corresponding O groups. Mean values for N.O were significantly lower with 74.1 and 76.7 HV2 compared with 81.7 and 82.5 HV2 for N.OT (*p* < 0.05). W.OT showed values of 81.6 and 81.8 HV2 compared with 74.7 and 74.0 HV2 for W.O.

The most homogeneous hardness distribution, expressed as ΔHV (max, min) of the mean values of a plane, was observed in W.OT (8.9 HV2), whereas N.OT (29.0 HV2) and N.O (28.1 HV2) showed significantly greater variability (*p* < 0.05). W.OT also achieved the highest mean hardness value for Plane A (81.8 HV2), while N.O showed the lowest value (73.0 HV2; *p* < 0.05). Although the mean hardness of N.OT (78.2 HV2) was significantly lower than that of W.OT (*p* < 0.05), it exceeded the values measured in both occlusally cured groups (W.O: 74.3 HV2). Post-cured groups showed a significantly higher overall mean value in Plane A than purely occlusally polymerized groups (*p* < 0.05).

In Plane A, post-curing resulted in significantly higher microhardness values at 8 of the 18 measurement points in the narrow-cavity groups (*p* < 0.05). In the wide-cavity groups, significant increases were observed at 15 of the 18 measurement points (*p* < 0.05). In the narrow-cavity configuration, the significantly enhanced measurement points were located within rows 2–5. In contrast, the wide-cavity configuration additionally exhibited significantly higher hardness values in row 1.

### 3.2. Plane B

**Figure 5:**
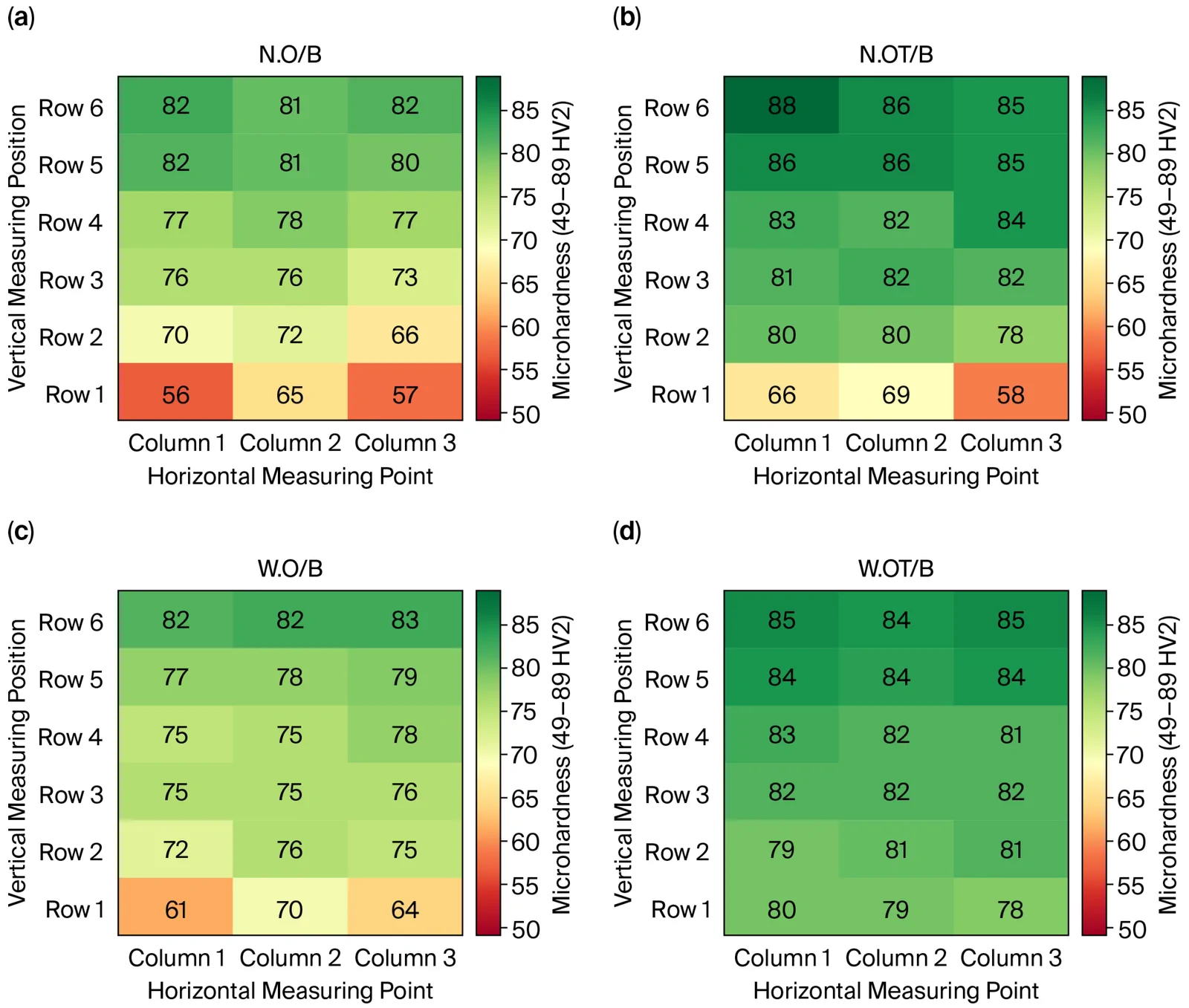
Heatmaps of mean Vickers microhardness (HV2) of the central Plane B for the four experimental groups (**a**): N.O, (**b**): N.OT, (**c**): W.O, (**d**): W.OT.

Similar trends were observed in Plane B, which runs through the central region of the restoration (Figure 5).

Microhardness values decreased in Plane B significantly (*p* < 0.05) from the occlusal to the crestal region in all groups. The smallest difference ΔHV(o,c) was similar to that in Plane A found in W.OT with 5.9 HV2 (84.9 vs. 79.0 HV2), whereas the largest difference was observed in N.O with 22.1 HV2 (81.7 vs. 59.6 HV2). N.OT showed a comparable ΔHV(o,c) of 22.0 HV2 (86.2 vs. 64.2 HV2) but maintained higher hardness values throughout the plane. W.O exhibited an intermediate difference of 17.1 HV2 (82.3 vs. 65.2 HV2).

The highest homogeneity was again observed in W.OT, with ΔHV(max,min) of 7.0 HV2 and the highest mean hardness value of 82.1 HV2. Greater variability was found in N.OT (30.1 HV2) N.O (26.4 HV2) and W.O (22.1 HV2) (*p* < 0.05). Despite this variability, N.OT showed a significantly higher mean hardness value (80.0 HV2) than both occlusally cured groups (N.O: 73.9 HV2; W.O. 75.2 Hv2; *p* < 0.05).

In Plane B, post-curing significantly increased microhardness at 12 of the 18 measurement points in the narrow-cavity groups (*p* < 0.05), whereas only 3 of the 18 measurement points showed significantly higher HV2 values in the wide-cavity groups (*p* < 0.05). In both cavity geometries, all measurement points located in row 1 exhibited significantly higher hardness values in the OT groups compared with the corresponding O group.

### 3.3. Plane C

**Figure 6:**
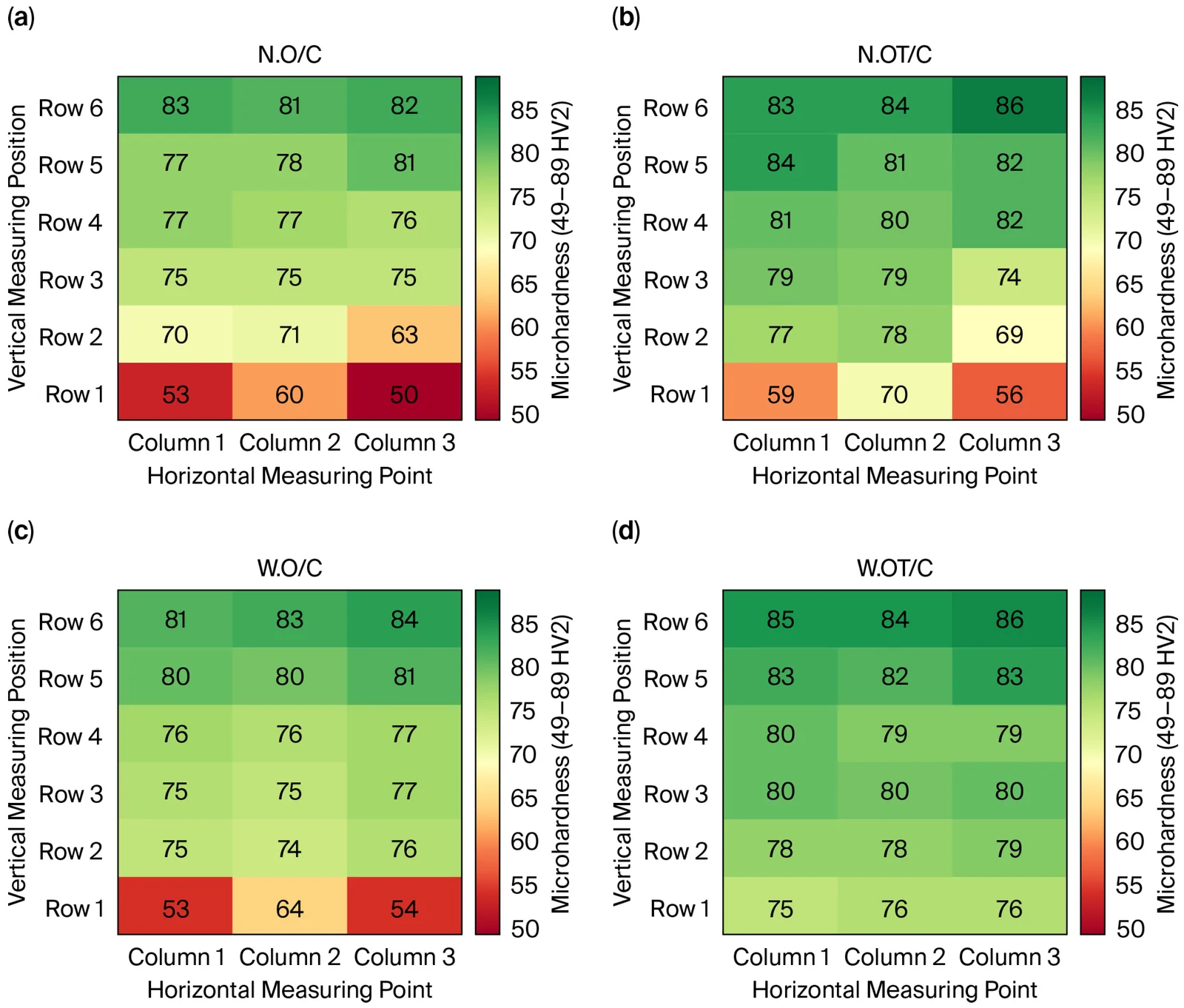
Heatmaps of mean Vickers microhardness (HV2) of the innermost Plane C for the four experimental groups (**a**): N.O, (**b**): N.OT, (**c**): W.O, (**d**): W.OT.

Plane C describes the innermost area of the restorations close to the pulp. In accordance with PPlanes A and B, plane C also displays differences between the differently hardened groups (Figure 6).

As observed in Planes A and B, the microhardness decreases from the occlusal to the crestal region. The smallest hardness gradient from occlusal to crestal was again measured in W.OT with ΔHV(o,c) = 9.4 HV2 (84.9 HV2 vs. 75.5 HV2), whereas the largest gradient occurred in N.O with 27.9 HV2 (82.2 HV2 vs. 54.3 HV2). Intermediate values were recorded for N.OT with 20.3 HV2 (84.5 HV2 vs. 64.2 HV2) and W.O with 25.8 HV2 (82.8 HV2 vs. 57.0 HV2).

W.OT demonstrated a significantly more homogeneous hardness distribution with (ΔHV(max,min) = 10.5 HV2 (85.5 vs. 75.0 HV2)) than the other groups (ΔHV(max,min) W.O = 31.0 HV2; ΔHV(max,min) N.O = 33.1; ΔHV(max,min) N.OT = 30.4 HV2) (*p* < 0.05).

The W.OT Group also achieved the highest mean hardness value of 80.2 HV2, followed by Group N.OT with 77 HV2, both of which were significantly higher than the control groups that were occlusally polymerized only (W.O: 74.6 HV2 and N.O: 72.5 HV2) (*p* < 0.05).

In Plane C, post-curing resulted in significantly higher microhardness values at 12 of the 18 measurement points in the wide-cavity groups compared with the corresponding occlusally cured groups (*p* < 0.05). In contrast, only 2 of the 18 measurement points exhibited significantly higher hardness values in the narrow-cavity groups following post-curing (*p* < 0.05).

Across all three planes, no measurement point showed significantly lower microhardness values in the OT group than the corresponding measurement point in the respective O group.

### 3.4. Total Hardness Values

Averaging microhardness values across all three planes showed the highest overall hardness value for Group W.OT with 81.4 HV2, whereas Group N.O exhibited the lowest overall hardness value with 73.5 HV2. The transdentally cured narrow-cavity Group N.OT showed higher overall hardness (78.4 HV2) than both purely occlusally cured groups (W.O: 74.7 HV2).

**Figure 7:**
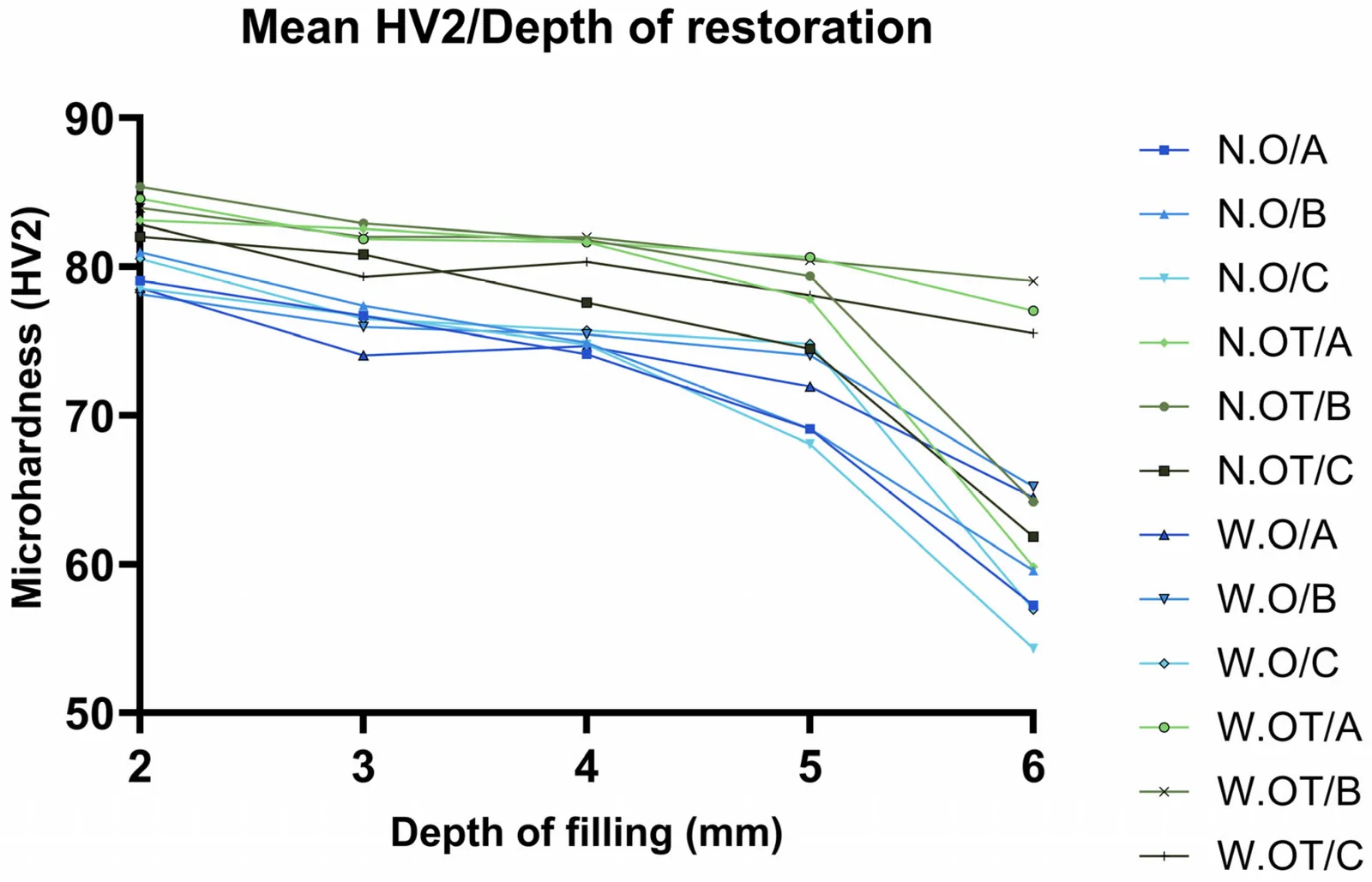
Mean microhardness in relation to the restoration depth illustrates the development of the average microhardness in HV2 of the filling layers in relation to the filling depth of the measured area.

Evaluating the mean HV2 values in relation to restoration depth, all groups showed average hardness values over 80 HV2 in the most occlusal row (Figure 7). In all groups, mean microhardness decreased significantly with increasing restoration depth (*p* < 0.05). This decrease was significantly more pronounced in O groups than in the corresponding OT groups (*p* < 0.05). Group W.OT exhibited the smallest overall reduction in hardness across restoration depth, whereas the N.O Group consistently showed the lowest hardness values across all planes. The deeper restoration layers showed significantly lower mean hardness values in all groups (*p* < 0.05).

**Figure 8:**
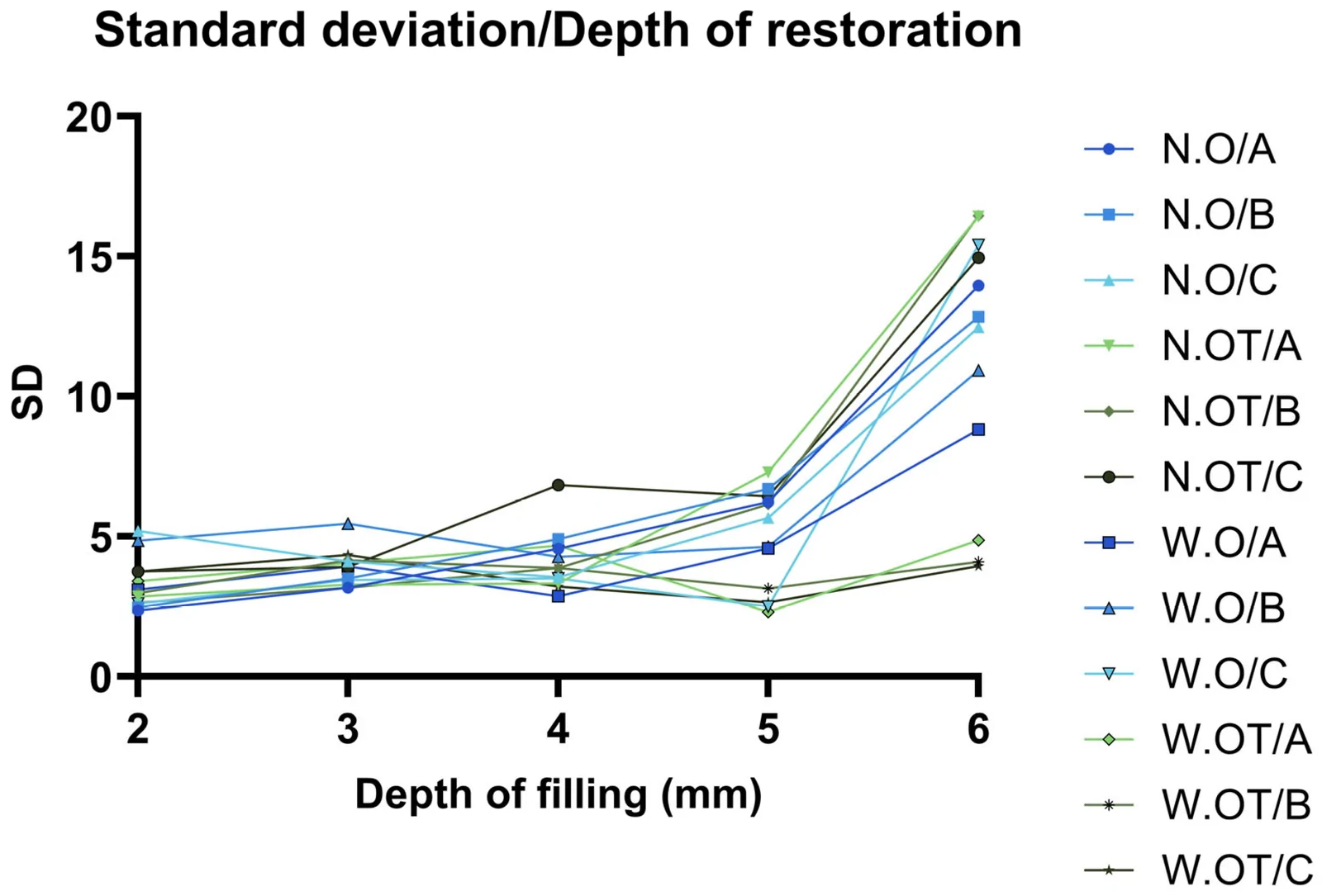
SD in relation to restoration depth shows the development of SD in relation to the filling depth of the measured area, broken down by the individual levels of the groups.

Standard deviation (SD) generally increased alongside restoration depth, reflecting greater variance in the measurements at deeper levels (Figure 8). The smallest elevation in SD and thus the highest consistency of the measured values in deep layers are shown by all levels of Group W.OT. While the levels of Group N.O also show low SD in higher layers, these increase significantly with increasing filling depth (*p* < 0.05). Group W.O also shows a significant increase in SD in Plane C in deep layers compared to occlusal layers (*p* < 0.05), with medium SD values in Planes A and B. The highest SD values in deep layers are shown by plane N.OT/A. Group N.O. shows high SD at high filling depth in all planes.

## 4. Discussion

Modern RBCs are the material of choice for most patients seeking esthetic and minimally invasive restorations [7,11]. Although long-term clinical data are encouraging and patient satisfaction is high, a certain degree of technique sensitivity remains critical for achieving optimal clinical performance [7,8,13]. Throughout the RBC placement workflow, two major factors contribute to this technique sensitivity, i.e., contamination management/field isolation and light curing [13,18,37,38]. While contamination management can be widely optimized using rubber dam isolation, light curing remains an underestimated and challenging factor for RBC longevity. Long-term RBC performance is mainly limited by degradation, secondary caries, and fractures, whereas all these scenarios are linked directly or indirectly to DoC and light curing [17,39]. Last but not least, long-term survival especially of bonded restorations is heavily dependent on operators and particularly their application protocol [13]. Since light curing protocols vary widely and the effects of post-curing are not yet fully understood, this study aims to address this gap.

The decision to use HV to determine the DoC was based on studies demonstrating a strong correlation between microhardness and the polymerization rate. A high DoC is expected to correlate with improved longevity and fracture resistance, whereas low DoC values may lead to accelerated degradation and restorative failure [40,41]. The advantage of HV measurements lies in the punctual prediction of DoC at specific points, allowing for the precise identification of weaker and stronger areas within the 3D volume of RBCs. Identifying these vulnerable regions could pave the way for optimizing clinical protocols and ensuring more uniform polymerization. Venus Diamond in the shade A3 serves as an ideal model system for this endeavor, given its frequent use in clinical practice and vast amounts of data being available regarding its average HV and degradation behavior [42].

Our results demonstrate that proximal post-curing significantly enhances HV across most of the tested planes. The observed increase in microhardness suggests that conventional occlusal curing alone may leave certain regions with incomplete polymerization, even when a high-performance LCU is used. Additional proximal irradiation appears to promote further conversion of residual monomers in these regions, resulting in a more uniform polymerization pattern throughout the restoration. Particularly, cervical areas, (Row 1), which are crucial for gap formation and secondary caries, demonstrated significant improvements in HV measurements in five out of six groups. This improvement is clinically relevant because these areas are particularly prone to marginal leakage, secondary caries and adhesive failure. Improving polymerization in these cervical areas may therefore have a greater clinical impact on fracture resistance and longevity of RBCs. Mean HV was in general found to decrease as the distance between the LCU and the composite increased, corroborating previous findings [24,26,27,41]. Besides increasing mean hardness, transdental post-curing significantly increased the homogeneous distribution of microhardness (ΔHV(max, min)), particularly in the W.OT Group. This finding might be of greater clinical relevance, as a restoration with high average HV but pronounced local minima remains only as strong as its weakest region, since localized areas of insufficient polymerization are more likely to fail by hydrolytic degradation, crack initiation and marginal failure. Post-curing therefore appears to help with compensating for areas that are systematically under-polymerized by occlusal curing alone. The only cervical area (Row 1) that did not improve in overall microhardness after post-curing was Group N, Plane A. This finding might be due to an accumulation of challenging conditions. The area has a large distance to the LCU. Furthermore, post-curing in this region was constrained by the presence of a closed proximal contact and a substantial thickness of enamel and dentin, which needs to be penetrated by the curing light. The persistent lower HV in Row 1 Group N/Plane A suggests that severely restricted access by tooth structure limits light penetration even when using state-of-the-art equipment. Thus, we conclude that cavity geometry and the resulting light accessibility greatly influences the effectiveness of both conventional light curing and proximal transdental post-curing. While Group N generally presented more challenging curing conditions, Group W benefited from occlusal light accessibility as well as direct exposure in post-curing due to its open proximal contact and thinner remaining tissue through which light had to penetrate.

Beyond the plane-specific homogeneity described above, post-curing also improved the consistency of polymerization across the entire restoration depth. Standard deviation of microhardness increased progressively with restoration depth in the occlusally cured groups, indicating that insufficient curing does not only lower the average DoC at depth, but also increases the variability and with this the unpredictability of microhardness in those areas. In comparison, post-curing led to comparatively low and stable SD values across all depths, especially in the W.OT Group, and makes polymerization outcome therefore more predictable. This might be especially relevant for long-term restoration durability.

Notably, proximal irradiation proved effective for post-curing in 48% of measured points as well as significantly enhancing HV in 83% of crestal areas. These results challenge earlier assumptions by demonstrating that relevant light transmission and polymerization can occur through both enamel and dentin [30]. However, this result might be due to higher irradiance of thepolymerization unit used in this study in comparison to earlier studies. For example, the LCU used here reaches up to 83% higher irradiance values and uses a light guide with a 25% wider aperture than LCUs used in previous studies [30]. Thus, this study also highlights the importance of using state-of-the-art LCUs with maximum irradiance and large apertures to ensure complete polymerization of the RBCs. The importance of this aspect increases in post-curing, where light transmission through enamel and dentin is required. While these findings underscore the necessity of high-irradiance and wide-aperture LCUs to overcome light attenuation in dental tissue, the choice of the matrix system might also influence polymerization of RBCs.

Metal matrices are generally considered superior to transparent matrices for Class-II restorations [43]. Consequently, the clinically widespread Palodent V3 metal matrix system was selected for this study. The primary limitation of metal matrices is the risk of shadowing, which can prevent adequate light curing of the RBC during polymerization [43]. Given the documented potential for metal matrices to induce shadowing, we hypothesized that Plane A would demonstrate significantly lower HV compared to Planes B and C in Group O (control). Interestingly, no significant shadow-casting effect was detected. We attribute this finding to the strictly perpendicular light application employed in our standardized setup. Thus, we conclude that metal matrices are theoretically prone to shadowing, but clinical under-polymerization is likely more a consequence of the angled LCU orientation common in posterior regions than the matrix material itself. Consequently, if strict perpendicular light curing cannot be guaranteed, proximal post-curing might help to polymerize regions near the matrix. Furthermore, previous studies have described reflective effects caused by metal matrices, potentially increasing light concentration within the cavity and thereby enhancing microhardness values [44,45]. Such effects may partly explain why no significantly lower HV values were observed in Plane A despite the theoretically increased risk of shadowing. If reflective effects on metal matrices indeed contribute to polymerization, additional transdental post-curing may be of particular relevance when transparent matrix systems are used, as these matrices cannot reflect light in a comparable way. However this hypothesis was not evaluated in the present study and requires further investigation.

Even though this study used standardized specimens in an extraoral setup, the observed positive effects of transdental curing might also be of clinical relevance. In clinical practice, successful light curing of RBCs can be particularly challenging for dental assistants due to poor accessibility of the respective region or lack of patient compliance. Thus, precise vertical irradiation of RBCs under these conditions is highly complex and the effects of shadowing must be taken into account. Since angled light curing can lead to insufficient polymerization, transdental post-curing may help to ensure homogenous hardening of RBCs even in hard-to-access regions. Overall based on the present in vitro findings we suggest considering transdental post-curing after removal of the metal matrix as a simple procedure that may help to improve RBC restoration quality in clinical contexts.

Although designed to closely simulate clinical conditions using a proximal contact simulation device and standardized conditions, this in vitro study is subject to several limitations. Firstly, the standardized extraoral experimental setup ensured high reproducibility but did not fully represent clinical conditions, including restricted intraoral access, moisture control, operator variability, and patient related factors. Secondly, our results cannot necessarily be generalized to other RBCs, as we only used one conventional nanohybrid RBC. This applies in particular to RBCs with different filler contents or Photoinitiator systems. This limitation also needs to be stated for the LCU, as only one high-performance LED polymerization lamp was used. Whether comparable results would be obtained with other LCUs with different irradiation profiles, beam homogeneity, or spectral emission remains unclear. Thirdly, specimens were not subject to a thermomechanical aging protocol or clinical performance evaluation, both of which may influence the clinical relevance of the observed differences in microhardness over time. Consequently, while the present findings provide mechanistic evidence for a beneficial effect of proximal post-curing, future studies should focus on long-term wear resistance, monomer elution, and clinical trials to confirm the hardness improvements in vivo.

## 5. Conclusions

Within the limits of this in vitro study, several conclusions can be drawn. Firstly, proximal post-curing significantly improved mean microhardness across the tested restorations. Secondly, the effectiveness of post-curing was influenced by cavity geometry. Wider cavities benefited considerably more than narrow ones, owing to their greater light accessibility and thinner residual tissue that light had to penetrate. Thirdly, proximal post-curing improved the homogeneity of microhardness distribution within the tested planes. Fourthly, post-curing lowered the SD of m easured microhardness across restoration depth, resulting in a more predictable polymerization outcome. Finally, no significant shadowing effects of matrices could be detected under the strict perpendicular light application in this study.

In summary, transdental post-curing appears to be a simple and potentially effective approach to achieve high polymerization rates and thus improve the quality of proximal RBCs. However, given the standardized in vitro conditions and material limitations discussed above, these findings require confirmation by clinical studies for final evaluation.

## Data Availability

The original contributions presented in the study are included in the article, further inquiries can be directed to the corresponding author.
